# Breast self examination and survival from breast cancer.

**DOI:** 10.1038/bjc.1992.385

**Published:** 1992-11

**Authors:** M. Le Geyte, D. Mant, M. P. Vessey, L. Jones, P. Yudkin

**Affiliations:** University of Oxford, Department of Public Health and Primary Care, Radcliffe Infirmary, UK.

## Abstract

The survival of 616 women aged 15-59 with breast cancer, 226 of whom had been taught and practised breast self examination (BSE) prior to diagnosis and 390 of whom had not, is reported. Six year survival rates were 73.1% in the BSE taught group and 66.1% in other women (P = 0.07).


 © Macmillan Press Ltd., 1992

SHORT COMMUNICATION

### Breast self examination and survival from breast cancer

M. Le Geyte, D. Mant, M.P. Vessey, L. Jones, & P. Yudkin

*University of Oxford, Department of Public Health and Primary Care, Gibson Building, Radcliffe Infirmary, Woodstock Road, Oxford OX2 6HE, UK.*

**Summary** The survival of 616 women aged 15–59 with breast cancer, 226 of whom had been taught and practised breast self examination (BSE) prior to diagnosis and 390 of whom had not, is reported. Six year survival rates were 73.1% in the BSE taught group and 66.1% in other women ($P = 0.07$).

In a previously reported investigation of 616 women with breast cancer included in a case-control study, we found an association between tumour stage at presentation and breast self examination (BSE) (Mant *et al.*, 1987). Women who had both practised and been taught BSE had more favourably staged tumours than other women. A number of other studies, summarised in the meta-analysis by Hill *et al.* (1988) support this observation and it now seems reasonable to conclude that, after adequate teaching and if carried out at reasonably frequent intervals, BSE does lead to earlier diagnosis. It is still unclear, however, whether this earlier diagnosis leads to a clinically important benefit in terms of reduced breast cancer mortality.

Overall breast cancer mortality in the UK Trial of the Early detection of Breast Cancer (UKTEDBC, 1988) was not reduced by mass education in BSE. However attendance at the BSE education session was limited and Locker *et al.* (1989) reported a marked difference in survival between attenders and non-attenders. This difference could be due to selection bias but Foster and Costanza (1984) have also reported from Vermont, USA that 5 year survival in women who perform BSE is 75% compared with 57% in non-performers, and that this difference could not be accounted for by the recorded characteristics of the women (other than cancer stage). The only other data available are from Japan, where Ota *et al.* (1989) reported a 5 year survival of 93% in women who had discovered their breast cancer by BSE compared with 85% in those who had made a fortuitous discovery. It is therefore of considerable interest that the cohort of women which we identified for our previous UK study has now been followed up for 6 years and we report here on whether the favourability in stage at diagnosis has been carried through to an improvement in survival from diagnosis.

### Method

Subjects were ever married women aged 15 to 59 years newly presenting with breast cancer at six London hospitals between September 1980 and December 1984. They were interviewd by nurses as part of a large case-control study of the relationship between oral contraceptives and breast cancer. The women were asked whether they practised BSE, whether they had ever been taught how to do BSE, how often they examined their breasts and who discovered the tumour. Breast cancer stage was established by an independent case not review.

Of 747 women interviewed, 44 were excluded because the cancer had been discovered other than by the woman herself. Staging information was available for 616 of the remaining 703 women who were followed up annually from entry into the study up to December 1989. Survival information was added to the computer file and manually verified: deaths from all causes are included. Loss to follow-up was small: only 17 women had been lost at the end of year 5 although follow-up of year 6 is still not complete for 44 women. A log rank test was used to assess the statistical significance of the difference between survival curves.

### Results

There were 130 deaths in the group that had never been taught BSE and 60 in the group that had been taught and practised BSE. The overall difference in survival is shown in Figure 1. The two survival curves began to diverge after year 1 with overall survival at the end of year 6 of 66.1% in the non-taught group and 73.1% in the BSE taught group. This is consistent with a lead time of about 18 months. The difference between the curves just fails to reach conventional levels of statistical significance ($P = 0.07$). Figure 2 shows survival according to stage; the survival benefit of taught BSE appears largely to be limited to patients with stage 1 disease, although the confidence intervals on the survival rates for stages 2–4 are wide.

### Discussion

In the women studied here the improvement in stage at diagnosis is carried through to increased survival at 6 years. There is a 7% probability that a difference as extreme as this would be seen by chance, even if there was no real difference in survival between the two groups. However, the more important uncertainty stems from the inability of a follow-up study to separate lead time from the effect of early treatment. The improvement in survival seen is from time of diagnosis

**Figure 1** Survival of women according to whether or not they had been taught BSE.

Correspondence: M.P. Vessey.
Received 11 October 1991; and in revised form 29 June 1992.

**Figure 2** Survival of women according to whether or not they had been taught BSE stratified by disease stage at diagnosis.

and, unlike the UKTEDBC, this study does not allow a direct measurement of the effect on mortality of early diagnosis resulting from teaching BSE. It is still possible that many of the cancers diagnosed in the BSE taught group had metastasised haematologically at diagnosis and the increase in survival seen reflects only advancement of diagnosis (i.e. lead time). However the fact that the survival benefit is essentially limited to patients with early stage tumours is consistent with an early treatment effect and while the survival benefit is sustained, the possibility that BSE is effective cannot be dismissed. As a definitive randomised trial now seems unlikely, perhaps we should accept that some women may extend their lives through BSE, although women should be made aware that if there is any benefit it is small in comparison to mammographic screening. The cost of BSE, particularly the anxiety experienced because of advancement of diagnosis and of false positive results in younger women, weighs heavily in formulating public health policy, and we still do not believe that BSE should be promoted as a means of screening for breast cancer.

### References

FOSTER, R.S. & CONSTANZA, M.C. (1984). Breast self examination and breast cancer survival. *Cancer*, **53**, 999.

HILL, D., WHITE, V., JOLLEY, D. & MAPPERSON, K. (1988). Self-examination of the breast: is it beneficial? Meta-analysis of studies investigating breast self examination and extent of disease in patients with breast cancer. *Br. Med. J.*, **297**, 271.

LOCKER, A., CASELDINE, J., MITCHELL, A., BLAMEY, R., ROEBUCK, E. & ELSTON, C. (1989). Results from a 7 year programme of breast self examination in 89,010 women. *Br. J. Cancer*, **60**, 401-405.

MANT, D., VESSEY, M.P., NEIL, A., MCPHERSON, K. & JONES, L. (1987). Breast self examination and breast cancer stage at diagnosis. *Br. J. Cancer*, **55**, 207.

OTA, J., HORINO, T., TAGUCHI, T. & 16 others (1989). Mass screening for breast cancer: comparison of the clinical stages and prognosis of breast cancer detected by mass screening and in out-patient clinics. *Jpn. J. Cancer Res.*, **80**, 1028.

UK TRIAL OF EARLY DETECTION OF BREAST CANCER GROUP (1988). First results on mortality reduction in the UK trial of early detection of breast cancer. *Lancet*, **ii**, 411.


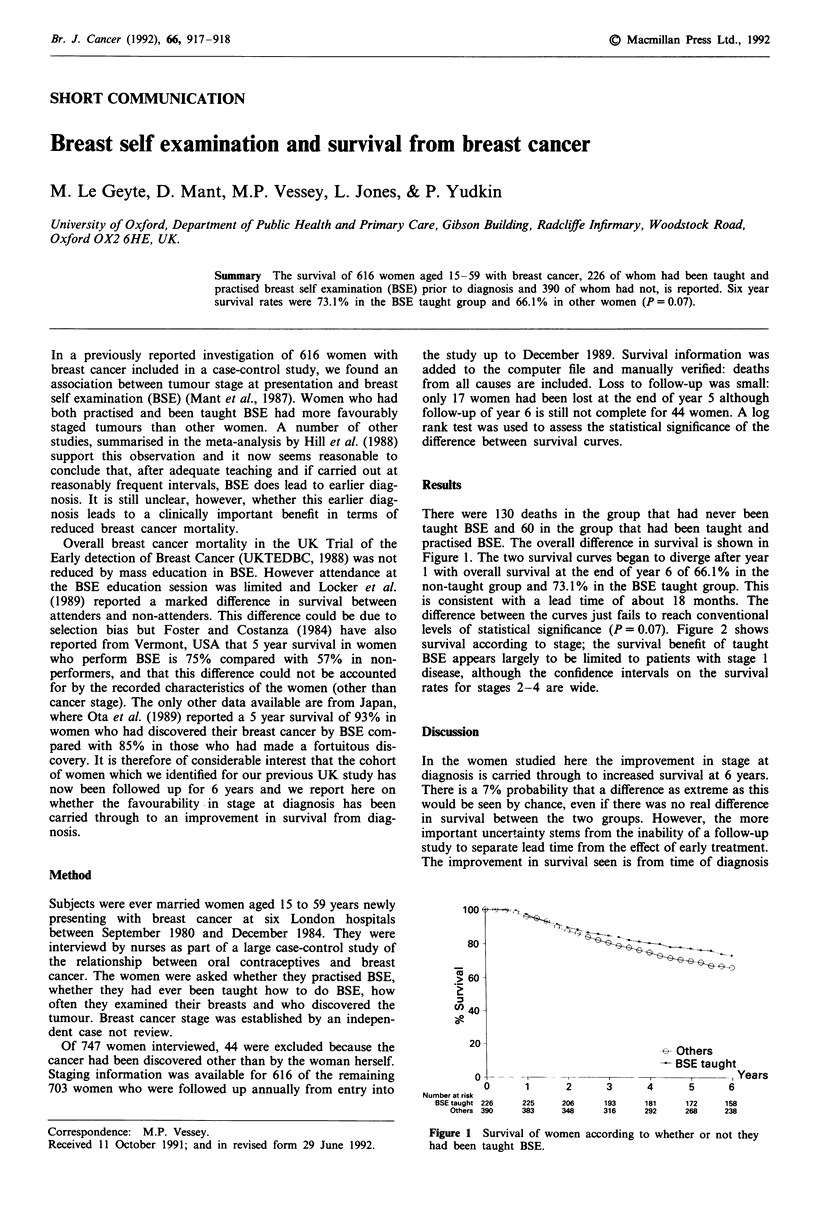

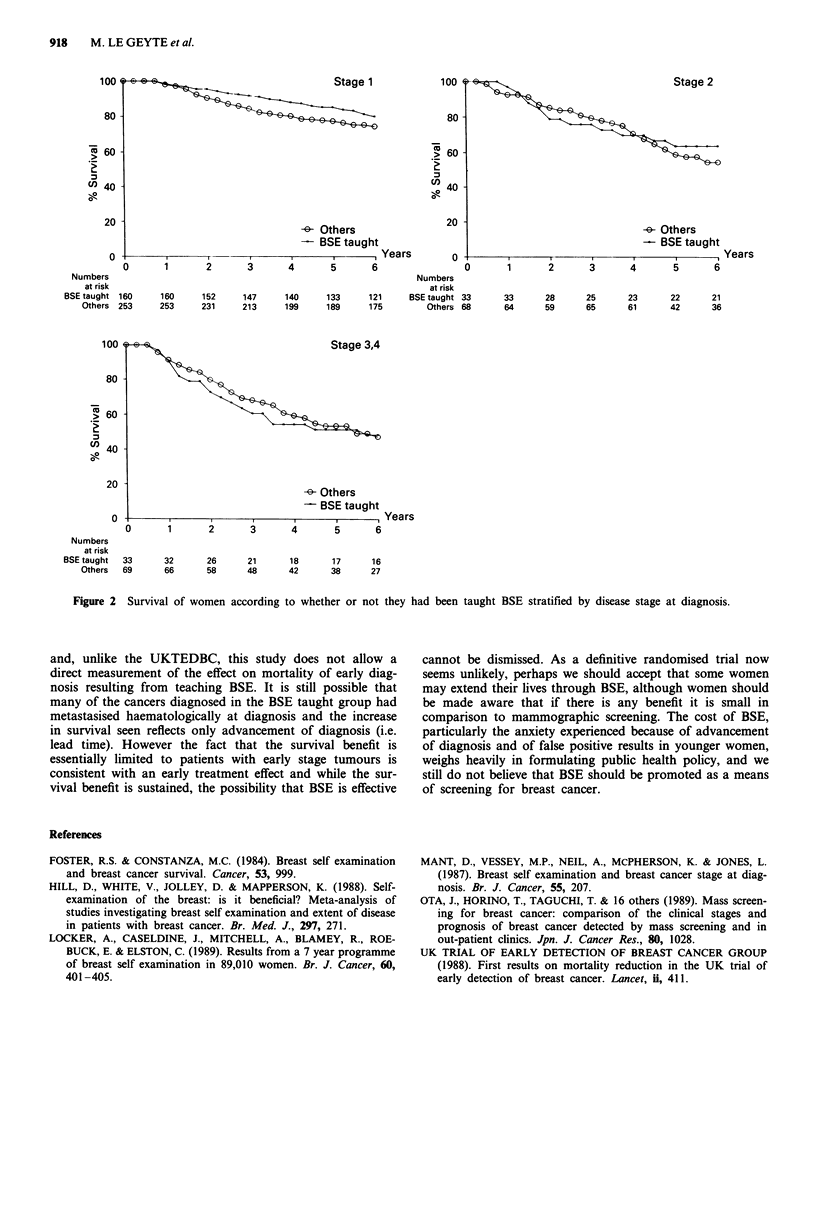


## References

[OCR_00229] Foster R. S., Costanza M. C. (1984). Breast self-examination practices and breast cancer survival.. Cancer.

[OCR_00233] Hill D., White V., Jolley D., Mapperson K. (1988). Self examination of the breast: is it beneficial? Meta-analysis of studies investigating breast self examination and extent of disease in patients with breast cancer.. BMJ.

[OCR_00241] Locker A. P., Caseldine J., Mitchell A. K., Blamey R. W., Roebuck E. J., Elston C. W. (1989). Results from a seven-year programme of breast self-examination in 89,010 women.. Br J Cancer.

[OCR_00245] Mant D., Vessey M. P., Neil A., McPherson K., Jones L. (1987). Breast self examination and breast cancer stage at diagnosis.. Br J Cancer.

[OCR_00250] Ota J., Horino T., Taguchi T., Ishida T., Izuo M., Ogita M., Abe R., Watanabe H., Morimoto T., Itoh S. (1989). Mass screening for breast cancer: comparison of the clinical stages and prognosis of breast cancer detected by mass screening and in out-patient clinics.. Jpn J Cancer Res.

